# Nutritional Interventions for Patients with Intermittent Claudication: Mechanisms, Clinical Implications, and the Case for Exercise-Adjunct Strategies: A Narrative Review

**DOI:** 10.3390/jcdd13100476

**Published:** 2026-09-22

**Authors:** Song-Young Park

**Affiliations:** Department of Physiology, College of Graduate Studies, Midwestern University, Downers Grove, IL 60515, USA; spark@midwestern.edu

**Keywords:** peripheral arterial disease, intermittent claudication, nutritional intervention, omega-3 fatty acids, L-arginine, carnitine, antioxidants, dietary nitrate, beetroot juice, exercise therapy

## Abstract

Peripheral arterial disease (PAD) is a common atherosclerotic condition affecting over 200 million people worldwide, and supervised exercise therapy remains the best-supported first-line treatment for claudication. However, real-world adherence to exercise programs is persistently low, largely because the ischemic leg pain that exercise is intended to treat is itself a primary barrier to participation. This narrative review synthesizes the mechanistic rationale and clinical trial evidence for five classes of nutritional intervention proposed as potential adjuncts to exercise in patients with intermittent claudication: omega-3 fatty acids/fish oil, L-arginine and other nitric oxide precursors, carnitine derivatives, antioxidant vitamins, and dietary nitrate (beetroot juice and related sodium nitrate/nitrite forms). Across these categories, mechanistic rationale is generally strong, but clinical translation to improved walking distance is inconsistent and depends heavily on route of administration, dose, and combination with exercise. Carnitine derivatives, particularly propionyl-L-carnitine, showed relatively consistent walking-distance improvement, though evidence remains limited and further confirmatory studies are needed. Dietary nitrate showed benefit primarily when combined with supervised exercise, suggesting potential as an adjunct rather than a standalone therapy, particularly for patients with limited exercise tolerance. L-arginine, despite a compelling mechanistic rationale, showed no benefit in the best-powered trial available. Antioxidant vitamins demonstrated route-dependent effects, with acute intravenous administration showing more promise than chronic oral supplementation. Overall, nutritional interventions for patients with intermittent claudication represent mechanistically diverse approaches with markedly uneven supporting evidence. Further well-designed, adequately powered trials are needed to clarify their efficacy, safety, and place alongside exercise in clinical practice.

## 1. Introduction

Peripheral arterial disease (PAD) is a common manifestation of systemic atherosclerosis, affecting well over 200 million people worldwide and imposing a substantial burden of morbidity, functional decline, and cardiovascular mortality [1,2]. The hallmark clinical presentation, intermittent claudication, is characterized by exercise-induced ischemic leg pain that resolves with rest, resulting from an inadequate matching of blood flow to metabolic demand in the affected limb. Beyond its vascular consequences, PAD is associated with progressive loss of ambulatory function, reduced quality of life, and markedly elevated risk of myocardial infarction, stroke, and death [2,3].

Current clinical practice guidelines, including the American Heart Association/American College of Cardiology (AHA/ACC) guideline on lower-extremity PAD, recommend supervised exercise therapy as first-line treatment for claudication, given consistent evidence from randomized controlled trials that structured walking programs improve maximal walking distance, pain-free walking time, and quality of life [4]. Exercise is understood to exert its benefit through a combination of mechanisms, including enhanced skeletal muscle oxidative metabolism, improved endothelial function, and favorable vascular remodeling in the affected limb.

Despite this strong evidence base, uptake of and adherence to exercise programs among PAD patients remains persistently low. In a study of 489 people with PAD in Chicago, most had not participated in supervised exercise therapy even 2–4 years after Medicare began covering it, and of those who participated, most completed fewer than half of the covered sessions; commonly cited barriers included the required copayment, being too time-consuming, too inconvenient, and lack of interest in treadmill exercise [5]. The very symptom that exercise is intended to treat, ischemic claudication pain, is also a primary barrier to engagement, and many patients are unwilling or unable to sustain a walking regimen that reliably provokes discomfort. As a result, a substantial proportion of patients with PAD do not receive adequate exposure to the treatment modality with the strongest evidence for functional benefit, underscoring a critical gap between guideline recommendations and real-world clinical practice.

This gap has motivated growing interest in nutritional and dietary interventions as potential adjuncts to exercise-based therapy in patients with intermittent claudication (claudicant). Many of the pathophysiological mechanisms underlying claudication—impaired nitric oxide bioavailability, oxidative stress, endothelial dysfunction, and skeletal muscle mitochondrial dysfunction—are, at least in principle, modifiable through nutritional means [6,7]. Agents such as omega-3 fatty acids, L-arginine and other nitric oxide precursors, carnitine derivatives, antioxidant vitamins, and dietary nitrate (commonly delivered via beetroot juice) have each been investigated for their capacity to target one or more of these mechanistic pathways, offering the theoretical appeal of a lower-burden intervention that does not depend on a patient’s willingness or ability to tolerate exercise-induced pain.

However, the evidence supporting these nutritional interventions is heterogeneous in both mechanistic rationale and clinical implications, spanning small mechanistic studies, underpowered pilot trials, and occasional larger randomized trials with inconsistent findings. To date, this literature has not been comprehensively synthesized in a way that connects the biological rationale for each nutritional strategy to its observed clinical effect on walking performance and vascular function in PAD. The purpose of this narrative review is therefore twofold: first, to summarize the mechanistic basis by which each major class of nutritional intervention—omega-3/fish oil, L-arginine and nitric oxide precursors, carnitine derivatives, antioxidant vitamins, and dietary nitrate—is hypothesized to benefit patients with intermittent claudication; and second, to critically synthesize the available clinical trial evidence for each, with particular attention to walking distance, vascular function, and quality-of-life outcomes. In doing so, this review aims to provide clinicians and researchers with an integrated resource to potentially guide the use of nutritional strategies as a complement to, or in some cases a substitute for, exercise-based therapy in the management of intermittent claudication who face persistent barriers to physical activity engagement.

## 2. Materials and Methods

### 2.1. Literature Search Strategy

A literature search was conducted across PubMed/MEDLINE, ScienceDirect, the Cochrane Library, and ClinicalTrials.gov to identify studies evaluating exercise, pharmacological, and nutritional interventions in patients with PAD. The search was last updated in August 2026. Reference lists of relevant reviews and meta-analyses were also hand-searched to identify additional primary studies not captured by the initial database search (backward citation searching).

### 2.2. Search Terms

Search terms combined the following concepts using Boolean operators (AND/OR): (i) Population—“peripheral arterial disease”, “peripheral artery disease”, “PAD”, “intermittent claudication”, “peripheral vascular disease”; (ii) Interventions—“exercise therapy”, “supervised exercise”, “omega-3”, “fish oil”, “n-3 polyunsaturated fatty acids”, “L-arginine”, “nitric oxide”, “carnitine”, “propionyl-L-carnitine”, “vitamin D”, “vitamin E”, “vitamin C”, “antioxidant”, “glutathione”, “beetroot juice”, “dietary nitrate”, “sodium nitrate”, “sodium nitrite”; and (iii) Study design—“randomized controlled trial”, “RCT”, “clinical trial”, “placebo-controlled”.

### 2.3. Inclusion and Exclusion Criteria

Inclusion criteria comprised: studies enrolling adults (≥18 years) with a confirmed diagnosis of PAD (typically defined by ankle-brachial index ≤ 0.90 and/or clinical diagnosis of intermittent claudication); interventional study designs, including randomized controlled trials, randomized crossover trials, and controlled pilot/feasibility studies; studies reporting on nutritional/dietary interventions; and studies published in English with no restriction on publication date to capture both foundational historical trials and contemporary studies. Exclusion criteria comprised: systematic reviews, meta-analyses, and narrative reviews (excluded from the primary evidence synthesis, though used to identify additional primary trials via reference mining and to contextualize findings in the Discussion); observational, cross-sectional, and case–control studies not involving an intervention; studies conducted exclusively in animal models or in vitro; conference abstracts and non-peer-reviewed sources; and studies in populations without confirmed PAD unless directly relevant to mechanistic rationale.

### 2.4. Study Selection, Data Extraction, and Methodological Limitations

Titles and abstracts were screened for relevance, followed by the full-text review of candidate studies. Screening, full-text review, and data extraction were performed by a single reviewer; formal dual-reviewer screening, a documented PRISMA flow diagram, and inter-rater agreement statistics were not employed, consistent with the narrative rather than systematic design of this review. No formal risk-of-bias or study-quality instrument was applied; instead, each table explicitly reports study design, sample size, and—where available—the analysis population (intention-to-treat [ITT], modified intention-to-treat [mITT], or per-protocol [PP]) underlying the reported result, so that readers can independently judge the strength of each finding. For each included study, the following fields were extracted where available: author/trial name, publication year and journal (or ClinicalTrials.gov identifier for registry-only entries), study design, sample size, intervention and comparator (including dose and duration), the primary or key outcome(s) as defined in the source publication, the analysis population, and the key numerical result(s) with associated statistical significance. Entries where a specific detail could not be confirmed in an accessible source are marked “NR” (not reported) rather than inferred. Studies are organized thematically by intervention class and, within each table, chronologically from oldest to newest (see Section 4, Limitations).

## 3. Results and Discussion

This section synthesizes the findings across five classes of nutritional intervention studied in patients with intermittent claudication. For each class, we first describe the proposed biological mechanism(s), then synthesize the clinical trial evidence for walking performance, vascular function, and quality-of-life outcomes, and close with directions for future research.

### 3.1. Omega-3 Fatty Acids/Fish Oil (Table 1)

#### 3.1.1. Mechanism(s)

Omega-3 polyunsaturated fatty acids (n-3 PUFAs), primarily eicosapentaenoic acid (EPA) and docosahexaenoic acid (DHA), are proposed to benefit vascular function in patients with intermittent claudication through several interrelated pathways. The most established mechanism is the enhancement of endothelial nitric oxide (NO) bioavailability: n-3 PUFAs activate endothelial nitric oxide synthase (eNOS) and reduce circulating levels of asymmetric dimethylarginine (ADMA), an endogenous eNOS inhibitor, thereby increasing endothelium-dependent vasodilation [8]. n-3 PUFAs also exert anti-inflammatory effects by reducing the production of pro-inflammatory cytokines and adhesion molecules (VCAM-1, ICAM-1, E-selectin) and by serving as precursors for specialized pro-resolving mediators (SPMs)—resolvins, protectins, and maresins—that actively promote the resolution of vascular inflammation rather than merely suppressing it [9,10]. Additional proposed mechanisms include antioxidant effects on low-density lipoprotein, favorable changes in membrane fluidity and ion channel function, and modest antithrombotic activity. In patients with intermittent claudication specifically, the omega-3 index (red blood cell EPA + DHA content) has been inversely associated with C-reactive protein, suggesting a direct link between omega-3 status and the systemic inflammatory burden characteristic of PAD [11].

**Table 1:** Omega-3/fish oil.

Study	Year	Design	N	Primary Endpoint	Analysis (ITT/PP/mITT)	Key Outcome/Result
Woodcock B, et al., Br Med J, 1984 [12]	1984	RCT	NR	NR	NR	Beneficial effect of fish oil on blood viscosity in peripheral vascular disease.
Ramírez-Tortosa C, et al. (Spanish Nutrition Study), Br J Nutr, 1999 [13]	1999	Randomized dietary intervention trial	NR	NR	NR	Olive oil- and fish oil-enriched diets modified plasma lipids and reduced LDL oxidative susceptibility in PVD patients.
Madden J, et al., Prostaglandins Leukot Essent Fatty Acids, 2007 [14]	2007	RCT	NR	NR (walking distance assessed; not explicitly designated primary in accessible source)	NR	Fish oil increased walking distance (not ABI); effect dependent on BMI and inflammatory genotype.
Altered Monocyte CD44 Expression in PAD Corrected by Fish Oil (Madden J, et al., Nutr Metab Cardiovasc Dis, 2009) [15]	2009	Clinical trial	NR	NR	NR	Monocyte CD44 MIF 480 ± 278 (PAD) vs. 336 ± 251 (control), *p* < 0.001 at the baseline. After 12 weeks of fish oil in PAD: CD44 reduced (480 ± 278 to 427 ± 262, *p* = 0.05); CD44v3 increased (0.15 ± 0.14 to 0.27 ± 0.23 OD units, *p* < 0.001)—altered monocyte markers returned toward the control levels.
OMEGA-PAD I Trial (Grenon et al., JAHA 2015; NCT01310270) [16]	2015	RCT, double-blind, placebo-controlled	80 (claudication)	Endothelial function (brachial artery FMD) at 1 month	All randomized patients analyzed (ITT not explicitly labeled)	FMD increased in the fish oil group (0.7 ± 1.8%) but not significantly different from the placebo (between-group *p* = 0.86); primary endpoint negative. Improved serum triglycerides and increased downstream n-3 PUFA-derived mediators.
Predictors of Omega-3 Index Change (OMEGA-PAD I secondary analysis), J Surg Res, 2017 [17]	2017	RCT secondary/ exploratory analysis	80 (40 fish oil/ 40 placebo)	Change in RBC omega-3 index (secondary analysis endpoint)	NR	Measured RBC fatty acid content via gas chromatography; change in omega-3 index calculated and baseline predictors of response examined.
Hammer A, et al., Br J Nutr, 2019 [18]	2019	RCT, double-blind, placebo-controlled	NR	Endothelial function (implied primary based on study aim; not confirmed verbatim)	NR	Assessed effects of n-3 PUFA supplementation on endothelial function in PAD patients.
OMEGA-PAD II Trial (J Surg Res, 2019) [19]	2019	RCT, double-blind, placebo-controlled	24	No single stated primary; co-primary: hs-CRP, FMD, WIQ, 6-min walk test	NR	No significant changes in hs-CRP, FMD, WIQ, or 6-min walk distance in the fish oil group. Omega-3 index and specialized pro-resolving mediators (SPMs) significantly increased vs. the placebo.
OMEGA-SPM-DOSE/ OMEGA-SPM-PAD Trials (NCT02719665) [20]	NR (registry; year not stated in source)	Pilot dose-finding study, Part 1a and 1b	Part 1a: 10 healthy + 10 PAD; Part 1b: n = 12	Metabolo-lipidomic/ pharmacokinetic profile (pilot; not a clinical functional endpoint)	NR	Measured metabolo-lipidomic profile, inflammation markers, and monocyte/macrophage function following novel SPM emulsion (n-3 PUFA metabolite) dosing.

#### 3.1.2. Clinical Implications

Despite a biologically plausible mechanism, clinical trial evidence for omega-3/fish oil supplementation in patients with intermittent claudication has been largely mixed to null for functional walking outcomes. In the OMEGA-PAD I trial, 80 patients with stable claudication received 4.4 g/day fish oil or the placebo for one month; flow-mediated dilation (FMD) increased numerically in the fish oil group but did not differ significantly from the placebo (between-group *p* = 0.86), despite significant increases in the omega-3 index [16,21]. The follow-up OMEGA-PAD II trial [19,22] extended supplementation to three months at the same dose but similarly did not demonstrate a clear functional benefit on walking ability. Older mechanistic studies reported favorable changes in blood viscosity and LDL oxidative susceptibility [12,13], and a study of BMI- and genotype-dependent responses found that fish oil increased walking distance in a manner dependent on body mass index and inflammatory genotype [14]—suggesting that responder subgroups may exist even where the average treatment effects are null. A monocyte CD44 expression study provided cellular-level evidence that fish oil can partially normalize markers of arterial disease, but this did not translate into a walking-distance endpoint [15]. The pilot OMEGA-SPM-DOSE/PAD trials represent an early attempt to test concentrated SPM metabolites rather than whole fish oil, but clinical efficacy data remain preliminary [20]. Taken together, the omega-3 evidence base in patients with intermittent claudication is characterized by consistent biochemical and inflammatory marker changes without a reliably corresponding improvement in walking distance or quality of life.

#### 3.1.3. Perspectives and Future Research

Future trials should consider: (1) larger, adequately powered samples specifically targeting walking distance and claudication onset time as primary rather than secondary endpoints; (2) stratification by inflammatory genotype or baseline omega-3 index to identify likely responders; (3) testing of purified, high-dose EPA formulations rather than mixed EPA/DHA products, given the contrasting results between purified-EPA and mixed-formulation trials in other cardiovascular populations; and (4) longer intervention durations, since vascular remodeling and membrane incorporation of n-3 PUFA may require months rather than weeks to translate into functional gains.

### 3.2. L-Arginine and Nitric Oxide Precursors (Table 2)

#### 3.2.1. Mechanism(s)

L-arginine is the obligate substrate for nitric oxide synthase (NOS), which converts L-arginine to NO and L-citrulline in vascular endothelium. Because plasma L-arginine concentrations under normal conditions already exceed the Michaelis–Menten constant (Km) of purified eNOS several-fold, the clinical benefit observed with L-arginine supplementation has been termed the ‘arginine paradox’ [23]. The leading explanation for this paradox is the competitive inhibition of eNOS by ADMA: in patients with atherosclerosis and PAD, ADMA levels are elevated up to two- to threefold above normal, and supplemental L-arginine is thought to restore the L-arginine/ADMA ratio sufficiently to overcome this inhibition and increase endothelium-derived NO [23,24]. A second contributing mechanism is competition between eNOS and arginase for the shared substrate pool; upregulated arginase activity, as seen in atherosclerotic vessels, diverts L-arginine toward urea/ornithine production and away from NO synthesis [25]. L-arginine supplementation is theorized to help overwhelm this competitive diversion by increasing substrate availability.

**Table 2:** L-arginine/nitric oxide precursors.

Study	Year	Design	N	Primary Endpoint	Analysis (ITT/PP/mITT)	Key Outcome/Result
Maxwell A, Anderson B, Cooke J (HeartBar), Vasc Med, 2000 [26]	2000	Double-blind, placebo-controlled RCT	41	NR (pain-free walking distance assessed; not explicitly labeled primary in accessible source)	NR	At 2-week follow-up: substantial reduction in leg pain and 66% increase in pain-free walking distance with L-arginine-fortified bar vs. placebo.
Pilot Dose-Finding Study (precursor to NO-PAIN; Oka RK, et al., Vasc Med, 2005) [27]	2005	RCT	80, 12 weeks	Absolute claudication distance (dose-ranging endpoint informing NO-PAIN sample size)	NR	Oral L-arginine doses of 0, 3, 6, or 9 g/day: no significant difference in absolute claudication distance across doses.
NO-PAIN Study (=Wilson AM, et al., Circulation, 2007; see also row below) [28]	2007	RCT, single-center	133 (claudication)	Change in absolute claudication distance (ACD) at 6 months (Skinner–Gardner treadmill protocol)	Modified intention-to-treat (mITT)	NO availability markers (FMD, vascular compliance, plasma/urinary nitrogen oxides, plasma citrulline) reduced or not improved vs. placebo despite raised plasma arginine. ACD improved less with L-arginine than the placebo (11.5% vs. 28.3%, *p* = 0.024)—primary endpoint favored the placebo.
Wilson AM, et al., Circulation, 2007 (NO-PAIN Study publication) [28]	2007	RCT, single-center, double-blind, placebo-controlled	133	Change in ACD at 6 months (same trial as above)	Modified ITT (mITT)	“L-arginine supplementation in PAD: no benefit and possible harm”. Long-term L-arginine did not increase NO synthesis or improve vascular reactivity; expected placebo effect on functional capacity was attenuated in the L-arginine group.

#### 3.2.2. Clinical Implications

In contrast to its mechanistic plausibility, clinical trials of oral L-arginine supplementation in PAD have been disappointing and in some cases concerning. The pivotal NO-PAIN study randomized 133 patients with claudication to oral L-arginine (3 g/day) or the placebo for 6 months; despite significantly raising the plasma arginine levels, the intervention did not improve—and in some NO-availability markers, appeared to worsen—flow-mediated dilation, vascular compliance, and nitrogen oxide measures relative to the placebo. Absolute claudication distance improved in both groups, but improvement in the L-arginine group was significantly less than the placebo (11.5% vs. 28.3%; *p* = 0.024), leading the authors to conclude “no benefit and possible harm” [28]. This dose-selection built directly on the group’s own prior work: a pilot dose-finding study [27] randomized 80 PAD patients with intermittent claudication to oral doses of 0, 3, 6, or 9 g/day of L-arginine for 12 weeks, using the Skinner–Gardner treadmill protocol and the Walking Impairment Questionnaire to establish the lowest effective dose [27]—this trial found no significant dose–response benefit, though a nonsignificant trend favored the 3 g dose, which was subsequently carried forward into the design of NO-PAIN. Notably, one of the more positive results in this category—the HeartBar trial—reported a 66% increase in pain-free walking distance and 23% increase in total walking distance after 2 weeks in patients taking two active bars/day (Maxwell, Anderson & Cooke, Vasc Med 2000) [26], with effects maintained through an 8-week open-label extension and accompanied by improvements in SF-36 general and emotional/social functioning scores, raising the possibility that any benefit observed was attributable to the multi-nutrient formulation rather than L-arginine alone. This concern is reinforced by a separate trial of the same HeartBar formulation: in 47 hypercholesterolemic (non-PAD) subjects receiving the identical 3.3 g L-arginine bar twice daily for 2 weeks, no favorable effect on flow-mediated dilation or platelet aggregation was observed [29], suggesting that the walking-distance benefit in claudicants may not be mediated through the proposed endothelial NO mechanism at all. Earlier work reported that L-arginine improved claudication symptoms [30], but this finding was not replicated in the larger, more rigorously controlled NO-PAIN trial.

#### 3.2.3. Perspectives and Future Research

Given the consistency of null-to-harmful findings in adequately powered trials, further isolated L-arginine supplementation in PAD populations may not be a productive avenue absent a compelling new rationale. More promising directions include: (1) investigating L-citrulline as an alternative NO precursor, since oral citrulline avoids first-pass hepatic arginase metabolism; (2) selecting patients based on the baseline ADMA/arginine ratio to identify the subset most likely to benefit; and (3) exploring whether L-arginine’s apparent benefit in multi-nutrient formulations reflects synergistic effects with co-administered antioxidants or B-vitamins, meriting study as combination rather than monotherapy.

### 3.3. Carnitine (Propionyl-L-Carnitine/Levocarnitine) (Table 3)

#### 3.3.1. Mechanism(s)

Carnitine’s proposed benefit in PAD centers on its essential role in mitochondrial fatty acid metabolism. L-carnitine shuttles long-chain fatty acids across the inner mitochondrial membrane via the carnitine palmitoyltransferase (CPT1/CPT2) system, enabling beta-oxidation and ATP production [31]. In ischemic skeletal muscle, PAD patients exhibit secondary carnitine deficiency along with the accumulation of acyl-CoA intermediates and acylcarnitines, metabolic derangements that correlate with reduced exercise performance [32,33]. Propionyl-L-carnitine (PLC), the propionyl ester of carnitine, is proposed to have additional anaplerotic activity, replenishing citric acid cycle intermediates via its propionyl group, and has been shown to increase muscle glycogen content and stimulate pyruvate dehydrogenase activity, effects not observed with equimolar L-carnitine alone [34]. Beyond its metabolic role, PLC has also been shown to act as an endothelium-dependent vasodilator, an effect mediated in part by prostaglandin synthesis rather than the nitric oxide pathway, and PLC’s antiradical/free-radical scavenging properties may further protect ischemic tissue.

**Table 3:** Carnitine (propionyl-l-carnitine/levocarnitine).

Study	Year	Design	N	Primary Endpoint	Analysis (ITT/PP/mITT)	Key Outcome/Result
Brevetti G, et al. (multiple trials, 1988–1999), JACC/ Eur Heart J/Circulation [34,35,36,37,38,39]	1988– 1999 (range)	Randomized, placebo-controlled trials (several)	NR (varies by trial)	NR (walking distance, varies by trial)	NR	Increased walking distance in claudicants across several early propionyl-L-carnitine/levocarnitine trials by this group.
European Multicenter Study on Propionyl-L-Carnitine, JACC, 1999 [35]	1999	RCT, multicenter, double-blind, placebo-controlled, stratified by baseline walking capacity	485, 12 months	Maximal walking distance (treadmill)	Both reported: ITT (primary, all randomized) and PP (target subgroup)	ITT: maximal walking distance +62 ± 14% (PLC) vs. +46 ± 9% (placebo), *p* < 0.05; no difference for initial claudication distance (ICD). PP analysis in target population (baseline walking ≤ 250 m, variability ≤ 25%) showed significantly greater PLC benefit for both MWD and ICD.
Hiatt WR, et al. (U.S./Russia), Am J Med, 2001 [40]	2001	Double-blind RCT, multicenter	155, 6 months	Peak walking time (treadmill)	NR (part of same phase III program as above; ITT likely but not independently confirmed)	Primary outcome peak walking time improved with PLC; also assessed claudication onset time, functional status, and safety.
L-Carnitine + Cilostazol vs. Cilostazol Alone (Goldenberg NA, et al., Vasc Med, 2012) [41]	2012	Multicenter, double-blind, placebo-controlled RCT	145 (mITT)	Peak walking time (mean ln ratio)	mITT population defined; primary result reported for per-protocol population	Peak walking time mean ln ratio 0.267 (L-carnitine + cilostazol) vs. 0.145 (cilostazol + placebo) in per-protocol population, *p* = 0.048; quality of life also improved.
Chinese Phase III Trial of Propionyl-L-Carnitine (Luo T, et al., Thromb Res, 2013) [42]	2013	RCT	239 (120/119), 4 months	Peak walking time (treadmill)	Per-protocol set (as reported)	Peak walking time increased 1.6 ± 1.6 min with PLC vs. placebo (*p* < 0.05, per-protocol set).

#### 3.3.2. Clinical Implications

Carnitine derivatives, particularly propionyl-L-carnitine, represent the most consistently positive nutritional intervention category identified in this review for improving walking performance in PAD. A European multicenter trial randomized 485 patients with intermittent claudication to PLC (1 g twice daily) or the placebo for 12 months; in the intention-to-treat population, maximal walking distance increased 62 ± 14% with PLC versus 46 ± 9% with the placebo (*p* < 0.05), with an even larger treatment effect in the pre-specified target population with more limited baseline walking distance [35]. This finding is consistent with the earliest RCT in this class: Brevetti et al. (Circulation, 1988) demonstrated increased walking distance with L-carnitine in a double-blind, cross-over study of patients with peripheral vascular disease [36], and was later corroborated by Allegra et al. (Ann Vasc Surg, 2008), who studied propionyl-L-carnitine specifically in Leriche–Fontaine stage II peripheral arterial obstructive disease [43]. A double-blind trial in a U.S./Russia cohort of 155 patients similarly found improvements in peak walking time with PLC (2 g/day) over 6 months [40]. A Chinese phase III trial (n = 239) replicated this benefit, with peak walking time increasing significantly more with PLC than the placebo (*p* < 0.05) [42]. Earlier trials across multiple publications (1988–1999) consistently demonstrated increased walking distance with levocarnitine or PLC [36,37,38].

Most recently, a trial adding L-carnitine to a cilostazol background found an incremental benefit on peak walking time over cilostazol plus the placebo, though this result requires careful interpretation: in the modified intention-to-treat population (n = 145), the primary endpoint—the mean ln-transformed ratio in peak walking time—did not reach significance (0.241 vs. 0.134, *p* = 0.076); only in the per-protocol population (n = 120) did the difference become significant (0.267 vs. 0.145, *p* = 0.048) [41]. Quality-of-life measures were also improved in the combination arm. This mixed clinical signal is bolstered by preclinical mechanistic data: in a rat hindlimb-ischemia model, the cilostazol/L-carnitine combination improved walking performance and increased capillary density in ischemic muscle relative to either agent alone [44], offering a biological rationale for pursuing the combination despite the missed primary endpoint in the human trial.

A 2021 Cochrane systematic review formally quantified the pooled effect across the PLC trial base: for absolute claudication distance, participants on propionyl-L-carnitine walked 50.86 m—approximately 26%—farther than those on the placebo, pooling data across trials including Brevetti 1995, Brevetti 1999, Coto 1992, Dal Lago 1999, Greco 1992, Hiatt 2001, Hiatt 2011, Luo 2013, and Signorelli 2006a, though heterogeneity across studies was very high (I^2^ > 85% for pooled outcomes) [45]. The review’s overall conclusion characterized the improvement in walking distance with PLC versus the placebo as mild to moderate, with moderate overall certainty of evidence [45]. An earlier, independent meta-analysis by Brass et al. situated this effect size in context: the magnitude of PLC’s effect on maximal walking performance was similar to other approved claudication drugs—pentoxifylline (44 m; 95% CI 14–74) and cilostazol (42 m; 95% CI 21–64)—but notably smaller than supervised exercise training (113 m; 95% CI 95–131) [46].

#### 3.3.3. Perspectives and Future Research

Given the relatively higher consistency of positive findings, carnitine derivatives—and PLC in particular—merit further investigation as an adjunct to first-line therapies. Priority directions include: (1) confirmatory modern trials using contemporary standardized treadmill protocols and patient-reported outcome measures, since much of the existing evidence predates current methodological standards; (2) formal investigation of carnitine combined with cilostazol or supervised exercise, given the promising per-protocol (though not intention-to-treat) signal from the existing combination trial, supported by preclinical angiogenesis data; (3) identification of a carnitine-deficiency biomarker or metabolomic signature that could be used to select patients most likely to respond, Hiatt and colleagues’ work characterizing carnitine-related metabolic alterations in patients with intermittent claudication provides an early foundation for this approach [39], and a Japanese trial specifically enrolling patients with intermittent claudication with sarcopenia and documented carnitine deficiency (JPRN-UMIN000016267) represents this patient-selection strategy already in progress; and (4) direct comparison of levocarnitine versus propionyl-L-carnitine, since the proposed anaplerotic advantage of the propionyl moiety has not been rigorously tested head-to-head in adequately powered walking-distance trials; the one small trial (n = 28) directly comparing the two forms found a nonsignificant 20 m advantage for PLC over L-carnitine in absolute claudication distance, rated low-certainty evidence [45].

### 3.4. Vitamin/Antioxidant Interventions

#### 3.4.1. Mechanism(s)

PAD is characterized by chronic oxidative stress arising from repeated cycles of ischemia-reperfusion in affected limb tissue during ambulation. Reduced blood flow followed by reperfusion generates excess reactive oxygen species (ROS), which both directly damage skeletal muscle myofibers and reduce nitric oxide bioavailability by oxidizing the eNOS cofactor tetrahydrobiopterin (BH4) to its inactive form dihydrobiopterin (BH2), a process that promotes eNOS ‘uncoupling’ and paradoxically converts the enzyme into an additional source of superoxide rather than NO [6]. This oxidative burden is compounded by compromised endogenous antioxidant defenses, including reduced manganese superoxide dismutase and glutathione peroxidase activity in PAD skeletal muscle [7]. Antioxidant vitamins and compounds are therefore hypothesized to interrupt this cycle through several routes: vitamin E and vitamin C act as direct free-radical scavengers; vitamin C additionally helps regenerate reduced BH4, supporting eNOS coupling; and glutathione, the primary endogenous intracellular antioxidant, directly neutralizes ROS generated during reperfusion while also supporting the redox state of the vascular endothelium.

#### 3.4.2. Clinical Implications

Clinical outcomes in this category diverge sharply based on route and duration of administration. Chronic oral antioxidant supplementation, whether single-agent vitamin E or multi-antioxidant combinations, has consistently failed to improve walking distance or claudication symptoms [47]. A 2-year multi-antioxidant trial (beta-carotene, vitamin C, pyridoxine, zinc, selenium) similarly found no difference in ankle-brachial pressure index or walking distance versus the placebo, although LDL cholesterol was modestly lower and serious adverse events were less frequent in the antioxidant group [48], with a secondary analysis of the same cohort finding improved depression scores at 2 years, suggesting possible benefits outside the primary vascular endpoint [49]. In contrast, acute, high-dose, intravenously administered antioxidants have shown more promising short-term physiological effects. Intravenous vitamin C prevented exercise-induced endothelial dysfunction and blunted oxidative stress markers after maximal exercise [50], and a related trial found intra-arterial vitamin C protective against ischemia-reperfusion-induced endothelial dysfunction [51]. Most notably, five days of twice-daily intravenous glutathione significantly increased the pain-free walking distance (196 ± 15 vs. 143 ± 11 m; *p* < 0.04) along with improved macrocirculatory flow and postischemic hyperemia [52], one of the few antioxidant interventions in this review with a direct, statistically significant walking-distance benefit. Combined oral vitamin E plus C reduced exercise-associated oxidative stress biomarkers without a reported walking-distance endpoint [53]. Allopurinol, a xanthine oxidase inhibitor tested for its antioxidant properties, significantly reduced uric acid but produced no change in walking distance, exercise capacity, or endothelial function [54], suggesting that xanthine oxidase inhibition alone is insufficient to translate into functional benefit.

#### 3.4.3. Perspectives and Future Research

The divergence between null chronic oral antioxidant trials and promising acute intravenous antioxidant trials suggest that route, dose, and timing relative to exercise-induced oxidative stress may be critical variables that have been underexplored. Future work should: (1) test whether the walking-distance benefit observed with intravenous glutathione is reproducible in larger, multi-center trials and whether benefits persist beyond the acute dosing period; (2) investigate oral antioxidant regimens specifically timed around exercise sessions, rather than continuous daily dosing, to more closely mimic the acute intravenous protocols that showed benefit; (3) explore targeted mitochondrial antioxidants (e.g., MitoQ) as a more mechanistically precise alternative to broad-spectrum vitamin supplementation; and (4) reconsider quality-of-life and mood outcomes as legitimate secondary endpoints in antioxidant trials, since these may represent a meaningful benefit independent of walking distance (Table 4). 

### 3.5. Beetroot Juice/Dietary Nitrate Supplementation

#### 3.5.1. Mechanism(s)

Dietary nitrate exerts its physiological effects through the nitrate–nitrite–nitric oxide pathway, an NOS-independent route to NO generation that is of particular relevance in PAD, where endogenous eNOS-derived NO production is already impaired. Ingested inorganic nitrate is partly concentrated in saliva via the enterosalivary circulation, where oral commensal bacteria reduce it to nitrite; swallowed nitrite is then absorbed into the circulation, where it can be further reduced to bioactive NO by deoxyhemoglobin, deoxymyoglobin, and other reductases under conditions of hypoxia and low pH [57,58]. This hypoxia-preferential activation is mechanistically well-suited to PAD, since ischemic skeletal muscle distal to arterial occlusion experiences precisely the low-oxygen, low-pH microenvironment that favors nitrite-to-NO conversion during exercise. The resulting NO promotes local vasodilation and may also improve mitochondrial efficiency, reducing the ATP cost of muscle force production [59]. Because this pathway bypasses eNOS entirely, dietary nitrate is theorized to restore NO-mediated vasodilation even in a dysfunctional endothelium—a distinct mechanistic advantage over L-arginine, which remains dependent on a functioning NOS enzyme.

#### 3.5.2. Clinical Outcome

Dietary nitrate is the most widely studied and recently investigated nutritional intervention class in this review, with evidence spanning beetroot juice, sodium nitrate, and sodium nitrite formulations. Across nearly all trial designs, nitrate supplementation reliably and reproducibly increased the plasma nitrate/nitrite concentrations and improved vascular physiological markers: an early crossover trial first demonstrated increased plasma nitrite and exercise tolerance with acute beetroot juice [60]; 8 weeks of oral sodium nitrate improved 6-min walk distance, peak calf blood flow, and vascular conductance [61]; and a related trial demonstrated improved blood pressure reactivity with the same regimen [62]. However, functional walking-distance benefits were inconsistent when nitrate was tested as a standalone acute or short-term intervention: a short-term coronary blood flow trial found no significant improvement in pain-free or peak treadmill walk time despite improved coronary hyperemic responses [63], and a sodium nitrite dose-escalation trial found no significant change in 6-min walk distance or quality of life despite improved flow-mediated dilation in the diabetic subgroup at the higher dose [64]. In contrast, the strongest and most clinically meaningful functional result in this entire review came from combining dietary nitrate with supervised exercise training: the NO-PAD trial found that 12 weeks of exercise plus beetroot juice significantly increased claudication onset time (+180.3 ± 46.6 s), 6-min walk distance (+53.4 ± 19.6 m), and peak walking time (+269.9 ± 195.3 s) compared to exercise plus the placebo (all *p* ≤ 0.05) [65], with a companion sub-study confirming that nitrate supplementation increased both the plasma and skeletal muscle nitrate/nitrite stores in this population [66]. A separate randomized crossover trial comparing nitrate-rich vegetables, beetroot juice, and a nitrate-depleted beetroot juice placebo in older adults with PAD similarly found that both active nitrate sources produced large increases in plasma nitrate and nitrite relative to the placebo, confirming that oral nitrate reliably elevates the systemic nitrate/nitrite pool regardless of source, although this trial’s primary focus was the acute pharmacokinetic response rather than walking-distance outcomes [67]. Sodium nitrite carried a modest tolerability burden, with headache and dizziness reported in 21–44% of patients at higher doses, though without clinically significant methemoglobinemia.

#### 3.5.3. Perspectives and Future Research

The dietary nitrate literature has matured rapidly and points toward a specific, testable hypothesis: that nitrate’s clinical value in PAD may lie chiefly in its capacity to potentiate exercise-based rehabilitation rather than to independently substitute for it. This is a particularly important distinction in the context of the low exercise adherence that motivates this review, since dietary nitrate may still hold value as a strategy to make a reduced, more tolerable dose of exercise more effective, even if it cannot replace exercise altogether. Future research directions include: (1) the ongoing multi-center BEET-PAD trial [68], which is well-positioned to clarify whether beetroot juice alone (without concurrent supervised exercise) produces a clinically meaningful 6-min walk distance benefit over a longer, 4-month follow-up; (2) formal dose-ranging and pharmacokinetic optimization of nitrate timing relative to exercise sessions; (3) trials in patients unable or unwilling to tolerate supervised exercise, to test whether nitrate supplementation alone can produce clinically meaningful, if more modest, functional gains; and (4) further mechanistic work using skeletal muscle biopsy or ^31^P magnetic resonance spectroscopy to confirm whether nitrate’s benefit in PAD operates primarily through vasodilation, improved mitochondrial efficiency, or both (Table 5). 

### 3.6. Summary of Cross-Category Findings

Considered together, the five nutritional intervention classes reviewed here show a consistent pattern: mechanistic rationale is strong across all categories, but clinical translation to improved walking distance is inconsistent and appears to depend heavily on route of administration, dose, and, most strikingly, combination with exercise. Carnitine derivatives appear to show relatively consistent monotherapy benefit in the available evidence, which may reflect their more direct correction of a well-characterized, disease-specific metabolic deficit (secondary carnitine deficiency), as opposed to the more diffuse anti-inflammatory or antioxidant mechanisms of other agents. Dietary nitrate shows the most promising trajectory when paired with exercise, offering a plausible bridge strategy for the substantial proportion of PAD patients who cannot or will not sustain supervised exercise therapy alone. L-arginine, in contrast, offers a cautionary example of a compelling in vitro mechanism that has not translated into a clinical benefit, and in at least one well-powered trial, trended toward harm—a reminder that the presence of a plausible pathway does not, on its own, justify continued clinical investment without confirmatory outcome data. The vitamin/antioxidant category illustrates that the same nutrient class can produce opposite conclusions depending on delivery method, with acute intravenous dosing showing more promise than chronic oral supplementation. Given the persistent barriers to exercise engagement described in the Introduction, the nutritional strategies most deserving of further clinical investment appear to be carnitine derivatives and dietary nitrate, both of which have shown the most consistent preliminary signals of benefits on walking performance in at least a subset of trial designs.

## 4. Limitations

### 4.1. Limitations of the Primary Evidence Base

Many of the trials identified in this review were small, single-center, and underpowered for walking-distance endpoints, particularly within the vitamin/antioxidant and L-arginine categories, where sample sizes frequently fell below 50 participants. Several foundational trials predate current methodological standards, and heterogeneity in dosing, formulation, treatment duration, and outcome measures (initial versus absolute claudication distance, peak walking time, 6-min walk distance) limits direct cross-trial comparison and precluded formal quantitative synthesis. Some nitrate trials were designed primarily to characterize acute pharmacokinetics rather than functional outcomes and should not be weighed equivalently to adequately powered walking-distance trials. Publication bias is also a plausible concern across all five categories, particularly for older and industry-sponsored trials.

### 4.2. Limitations of the Review Methodology

This review was conducted as a narrative rather than a systematic review. Study identification and selection were performed by a single reviewer without formal dual-screening, a pre-registered protocol, or a documented risk-of-bias assessment tool, meaning that completeness and reproducibility cannot be guaranteed to systematic-review standards. The search, while supplemented by reference-list mining, was not exhaustive and may have missed relevant non-English-language or unindexed trials. No formal quality grading was applied to individual studies, and because no meta-analysis was performed, conclusions about the relative strength of evidence across categories reflect qualitative synthesis rather than pooled effect estimates.

### 4.3. Implications for Future Research

A formal systematic review, and where sufficient homogeneous trials exist (most plausibly for carnitine derivatives and dietary nitrate), a quantitative meta-analysis would allow for a more rigorous synthesis of effect sizes and moderating variables such as baseline disease severity and dose. Standardization of walking-distance outcome measures across future PAD nutrition trials would improve cross-study comparability, and given the adherence problem motivating this review, future trials should more consistently report adherence and tolerability alongside walking-distance endpoints.

## 5. Conclusions

Supervised exercise therapy remains the best-supported treatment for PAD-related claudication, but poor real-world adherence due to exercise-induced pain, inconvenience, and cost leaves many patients without access to it. This review examined whether nutritional interventions could help fill that gap, either as an adjunct to exercise or as a partial alternative.

Across the five intervention classes reviewed, no single nutritional strategy emerged as a clear standalone replacement for exercise, and the overall evidence is too heterogeneous in design and quality to support firm rankings between categories. Carnitine derivatives, particularly propionyl-L-carnitine, showed a comparatively more consistent signal of walking-distance benefit across trials, which is at least plausible given their proposed role in addressing a metabolic deficit described in ischemic skeletal muscle, though this remains a proposed mechanism rather than a confirmed explanation for the clinical findings. Dietary nitrate appeared to show more consistent beneficial effects when combined with supervised exercise than when used alone, which may suggest a role in augmenting exercise-related functional benefits, though this pattern comes from a small number of trials and would benefit from replication before being treated as established. L-arginine offers an instructive example of the limits of mechanistic reasoning alone: despite a plausible rationale, the most rigorously designed trial in this category found no benefit and a signal potentially suggesting harm, a reminder that plausible mechanisms do not always predict clinical outcomes. The antioxidant category suggests a related, though tentative, pattern: chronic oral supplementation trials largely failed to show benefit, while smaller studies of acute intravenous dosing showed more encouraging short-term results, a route-and-timing distinction that seems worth further investigation rather than one that can yet be treated as established.

Taken together, carnitine derivatives currently show the most consistent, though still limited, evidence of benefit among the interventions reviewed here, and dietary nitrate appears to hold the most promise as a potential adjunct to exercise rather than as a substitute for it. The trial evidence to date does not support L-arginine monotherapy as a promising direction for further investment, and antioxidant research may be better directed toward acute, targeted delivery approaches than toward further chronic supplementation trials, though these are implications drawn from a limited evidence base rather than conclusions that should guide clinical practice.

## Figures and Tables

**Table 4:** Vitamin/antioxidant.

Study	Year	Design	N	Primary Endpoint	Analysis (ITT/PP/mITT)	Key Outcome/Result
Livingston & Jones, Lancet, 1958 [55]	1958	Early clinical trial (randomization/blinding NR by modern standards)	NR	NR	NR	Historical trial of vitamin E for treatment of intermittent claudication.
Williams, Clein & Macbeth, Can Med Assoc J, 1962 [56]	1962	Preliminary clinical report	NR	NR	NR	Early/preliminary evidence for alpha-tocopherol (vitamin E) in intermittent claudication.
Leng, Lee, Fowkes et al., Vascular Medicine, 1997 [48]	1997	RCT, 2-year follow-up	120	Ankle-brachial pressure index (ABPI) and walking distance	Intention-to-treat (as reported)	No significant difference in ABPI or walking distance vs. placebo; LDL cholesterol significantly lower (108.0 vs. 120.1 mg/dL, *p* < 0.05); fewer serious adverse events (21.8% vs. 40.0%).
Impact of Antioxidant Therapy on Anxiety/Depression (secondary analysis of Leng et al., J Nutr Environ Med, 1998) [49]	1998	RCT secondary analysis	120	Depression/anxiety scores (secondary analysis endpoint, not the parent trial’s primary endpoint)	NR	Depression scores significantly lower in the antioxidant group after 2 years; trend toward less anxiety.
Wijnen, Coolen, Vader et al., J Surg Res, 2001 [53]	2001	Clinical trial, 4-week supplementation	NR	Exercise-associated oxidative stress markers (implied primary)	NR	Combined oral vitamin E (200 mg) + vitamin C (500 mg) daily reduced exercise-associated oxidative stress in claudicant patients.
Silvestro, Scopacasa, Oliva et al., Atherosclerosis, 2002 [50]	2002	RCT	31 claudicant patients	Exercise-induced endothelial dysfunction (FMD) (implied primary)	NR	IV vitamin C infusion before exercise prevented exercise-induced endothelial dysfunction (FMD decline) and blunted the rise in oxidative stress markers (TBARS, sICAM-1) after maximal exercise.
Arosio, De Marchi, Zannoni et al., Mayo Clin Proc, 2002 [52]	2002	RCT, double-blind, placebo-controlled	40 (Fontaine stage II)	Pain-free walking distance (implied primary)	NR	IV glutathione increased pain-free walking distance (196 ± 15 vs. 143 ± 11 m; *p* < 0.04); improved macrocirculatory flow (*p* < 0.002) and postischemic hyperemia (*p* < 0.005).
PoleStriding Exercise and Vitamin E for Management of PVD (Collins EG, et al., Med Sci Sports Exerc, 2003) [47]	2003	RCT, 4-arm (PoleStriding, vitamin E, combined, control)	NR	Leg pain and treadmill walking duration (implied primary; not explicitly labeled)	NR	Vitamin E did NOT significantly improve leg pain (*p* = 0.35) or treadmill walking duration (*p* = 0.36); PoleStriding exercise alone was effective (*p* < 0.001).
Intra-arterial Vitamin C Prevents Endothelial Dysfunction Caused by Ischemia-Reperfusion (Pleiner J, et al., Atherosclerosis, 2008) [51]	2008	RCT	26 healthy + 8 PAD patients	Forearm blood flow response to ischemia-reperfusion (implied primary)	NR	Intra-arterial vitamin C prevented ischemia-reperfusion-induced endothelial dysfunction, measured via forearm blood flow responses.
Robertson & Struthers, Can J Cardiol, 2016 [54]	2016	Double-blind RCT, 6-month treatment	50	Change in exercise capacity on treadmill testing at 6 months (confirmed primary endpoint)	NR (not explicitly stated as ITT/PP in accessible abstract)	Allopurinol significantly reduced uric acid (52.1%, *p* < 0.001); NO significant change in pain-free/maximum walking distance, exercise capacity, or endothelial function—primary endpoint negative.

**Table 5:** Beetroot juice/dietary nitrate supplementation.

Study	Year	Design	N	Primary Endpoint	Analysis (ITT/PP/mITT)	Key Outcome/Result
Kenjale AA, et al., J Appl Physiol, 2011 [60]	2011	Randomized, open-label, crossover	NR	Exercise tolerance/ time to claudication (implied; foundational study)	NR	Foundational study: BR juice increased plasma nitrite, exercise tolerance, and decreased gastrocnemius fractional O_2_ extraction vs. the placebo.
SONIC Trial (Phase IIa), NCT01401517 [same underlying study as Mohler ER 3rd 2014, see row below] [64]	2014 (same trial as Mohler 2014)	Safety/PK/ tolerability trial, dose escalation	NR	Safety and tolerability (Phase IIa primary focus); FMD, 6MWD, QoL as efficacy measures	NR	Assessed FMD, 6-min walk distance, and QoL (WIQ/SF-36) changes at 10 weeks vs. baseline.
Mohler ER 3rd, et al., Vasc Med, 2014 (published results of the SONIC trial, NCT01401517) [64]	2014	Randomized, placebo-controlled, dose-escalation (1:1:1)	55, 10 weeks	Safety, walking distance (6-min walk), and endothelial function —confirmed via published title	NR	No significant change in 6-min walk test or QoL vs. placebo; FMD stable/improved at 80 mg dose in diabetics; well-tolerated; headache/dizziness in 21–44%.
Allen JD, et al., Nitric Oxide, 2018 [61]	2018	Randomized, placebo-controlled	21, 8 weeks	6-min walk distance/ peak calf blood flow (implied primary)	NR	Elevated plasma nitrate/nitrite; improved 6-min walk (387 ± 90 to 425 ± 82 m), peak calf blood flow, and peak calf vascular conductance (*p* < 0.05).
Woessner M, et al. (NO-PAD Trial), Circ Res, 2018, NCT01684930 [65]	2018	Randomized, double-blind, controlled pilot RCT	30, 12 weeks	Exercise capacity (claudication onset time, peak walk time, VO2peak) and functional capacity (6-min walk distance)—confirmed co-primary aims	NR (pilot study; ITT/PP not explicit in accessible abstract)	EX + BR significantly increased claudication onset time (+180.3 ± 46.6 s, *p* ≤ 0.05), 6-min walk distance (+53.4 ± 19.6 m, *p* ≤ 0.05), and peak walking time (+269.9 ± 195.3 s, *p* ≤ 0.01) vs. EX + PL.
Pekas EJ, et al., Am J Physiol Regul Integr Comp Physiol, 2021/ Microvasc. Res., 2023 [69,70]	2021	Randomized crossover design	10 (PAD)	Skeletal muscle microvascular/ endothelial function and walking capacity (implied primary)	NR	Moderate-dose, body-mass-normalized dietary nitrate improved skeletal muscle microvascular function, endothelial function, and walking capacity.
van der Avoort CMT, et al. (acute nitrate trial), Int J Sport Nutr Exerc Metab, 2021 [corrected from “Kim DJ”—see verification memo] [67]	2021	Randomized, double-blind, placebo-controlled crossover	18 (age 73 ± 8)	Plasma nitrate/nitrite response (acute pharmacokinetic-type endpoint, implied)	NR	Plasma nitrate and nitrite increased 14-fold and 7-fold, respectively, after BRJ (not seen with the nitrate-depleted placebo). NOTE: This specific fold-change figure was not independently re-confirmed against the published abstract in the verification pass and should be checked against the full text.
Hughes WE, et al., Nutr Metab Cardiovasc Dis, 2022, NCT01983826 [62]	2022	Randomized, placebo-controlled	21 (13 NaNO_3_/ 8 placebo), 8 weeks	Blood pressure reactivity indices (implied primary)	NR	Improved blood pressure reactivity indices in PAD patients with elevated BP and exaggerated pressor responses.
Piknova B, et al. (sub-study of NO-PAD trial), Physiol Rep, 2022 [66]	2022	RCT sub-study, double-blind, parallel-design	Sub-study of n = 30 parent trial	Skeletal muscle nitrate/nitrite content (sub-study-specific, mechanistic)	NR	First study of BRJ effects on skeletal muscle nitrate/nitrite in PAD; acute nitrate increased both plasma and muscle nitrate (*p* < 0.01), correlated (r = 0.63, *p* < 0.01).
Kim DJK, et al. (short-term nitrate, coronary blood flow), Frontiers Nutr, 2024 (NCT02553733) [63]	2024	Randomized, double-blind, placebo-controlled crossover	11 (52–80 yr)	Peak treadmill time AND peak coronary hyperemic response to plantar flexion exercise (dual confirmed primary endpoints)	NR	No significant improvement in pain-free/peak treadmill walk time vs. placebo; improved coronary hyperemic and blood pressure responses during calf ischemia; safe and well-tolerated.
Effect of Beetroot Juice on Coronary Blood Flow and Walking Performance in PAD, NCT02553733 [same trial registry as Kim DJK 2024 above] [63]	2024 (same trial as Kim DJK 2024)	Phase 2, randomized, double-blind crossover	12 (per registry results ~10/arm)	Peak treadmill time (Gardner protocol) —confirmed via ClinicalTrials.gov results posting	NR	Assessed treadmill walking (day 4) and coronary/lower-leg vasodilator responses (days 5–7); peak treadmill time was the registered primary outcome.
Eight Weeks of Nitrate Supplementation Improves Blood Flow…, Am J Physiol Heart Circ Physiol [71]	NR (year not stated in source)	Randomized, placebo-controlled, 8 weeks	NR	Exercising forearm blood flow (implied primary)	NR	Exercising forearm blood flow improved (141 ± 17 to 172 ± 20 mL/min); mean arterial pressure response reduced (13 ± 1 to 9 ± 1 mmHg, *p* < 0.05).
BEET-PAD Trial, NCT05624125 (ongoing) [68]	Ongoing (registered ~2022–2023)	Multi-centered, double-blind RCT	NR (ongoing; target N not stated in source)	4-month change in 6-min walk distance —confirmed explicit primary outcome via ClinicalTrials.gov	Planned—trial ongoing; analysis population not yet reported	Primary aim: change in 6-min walk distance at 4 months; secondary: gastrocnemius muscle perfusion (MRI) and muscle health.

## Data Availability

No new data were created. All data discussed are available in the cited publications.
